# Adaptation of utility functions to reward distribution in rhesus monkeys

**DOI:** 10.1016/j.cognition.2021.104764

**Published:** 2021-09

**Authors:** Philipe M. Bujold, Simone Ferrari-Toniolo, Wolfram Schultz

**Affiliations:** Department of Physiology, Development and Neuroscience, University of Cambridge, Cambridge CB2 3DY, United Kingdom

**Keywords:** Choice, Prospect theory, Reference, Risk, Standard deviation

## Abstract

This study investigated how the experience of different reward distributions would shape the utility functions that can be inferred from economic choice. Despite the generally accepted notion that utility functions are not insensitive to external references, the exact way in which such changes take place remains largely unknown. Here we benefitted from the capacity to engage in thorough and prolonged empirical tests of economic choice by one of our evolutionary cousins, the rhesus macaque. We analyzed data from thousands of binary choices and found that the animals' preferences changed depending on the statistics of rewards experienced in the past (up to weeks) and that these changes could reflect monkeys' adapting their expectations of reward. The utility functions we elicited from their choices stretched and shifted over several months of sequential changes in the mean and range of rewards that the macaques experienced. However, this adaptation was usually incomplete, suggesting that – even after months - past experiences held weight when monkeys' assigned value to future rewards. Rather than having stable and fixed preferences assumed by normative economic models, our results demonstrate that rhesus macaques flexibly shape their preferences around the past and present statistics of their environment. That is, rather than relying on a singular reference-point, reference-dependent preferences are likely to capture a monkey's range of expectations.

## 1 Introduction

It is generally assumed that adaptation to current statistical distributions of physical events enables efficient processing by systems with limited dynamic range. Adaptation improves discrimination, increases effective working range, and maximizes information processing. The phenomenon has been particularly well investigated in biological systems; sensory neurons of all modalities adapt their sensitivity to the environment (Barlow, 1961; Laughlin, 1981; Heeger, 1992; Fairhall, Lewen, Bialek, & De Ruyter van Steveninck, 2001; Hosoya, Baccus, & Meister, 2005; Maravall, Petersen, Fairhall, Arabzadeh, & Diamond, 2007). Rewards and economic objects are no exception: although standard economic theories deal with economic outcomes as if their utility were independent of anything other than the object itself (Von Neumann and Morgenstern, 1944; Luce, 1959), empirical studies of economic choice reveal a number of phenomena suggestive of adaption, including the reference dependency of Prospect Theory (henceforth PT, Kahneman & Tversky, 1979; Tversky & Kahneman, 1992) and the violation of the independence axiom (Tversky & Simonson, 1993; for review, see Rieskamp, Busemeyer, & Mellers, 2006).

Specifically, PT posits that we optimize our decisions by calculating the value of our choices relative to a reference-point (Tversky & Kahneman, 1986). That is, rather than objectively evaluating the outcome of our choices, we perceive our options as gains or losses depending on what we are expecting: if an outcome is better than our reference, we treat it as a gain; if it is worse, we treat it as a loss. Mathematically, PT represents this behavior with an S-shaped value (or utility) function where the subjective value of gains and losses is given by concave and convex parts of the function, respectively. This has important behavioral consequences, particularly for risky-decision-making, as this normative (utility) framework predicts that people's tendency towards risk averse or risk-seeking decisions depends on their perception of outcomes as being gains or losses.

While the idea of reference-dependence has been readily adopted by modern decision theory (Rabin, 2000; Wakker, 2010), economists are still unclear about how reference points form (Barberis, 2012). In PT, Kahneman and Tversky abstractly define reference-points as exogenous from the decisions being made. That is, the reference point is not directly explained by PT and can be shaped by “*aspirations, expectations, norms, and social comparisons”* (A. Tversky & Kahneman, 1991, p.157). Alternatively, recent economic models consider reference points an epiphenomenon of the way our mind adapts to the statistics of the task at hand (Delquié & Cillo, 2006; Köszegi & Rabin, 2006; Sugden, 2003) - a framework more in line with the findings that, far from being restricted to human reasoning, reference-dependence is a homogeneous feature of primate decision-making and the brain (Carandini & Heeger, 2012; Louie, Khaw, & Glimcher, 2013; Padoa-Schioppa, 2009; Santos & Rosati, 2015; Tremblay & Schultz, 1999). Along these lines, one particularly interesting proposal from the epiphenomenon framework is that of range-dependent utility, or RDU (a play on reference-dependent utility; see Kontek & Lewandowski, 2018). Inspired by psychology's *range-frequency theory* (Parducci, 1965, Parducci, 2012) and neurobiology's *efficient-coding hypothesis* (Laughlin, 1981; Summerfield & Tsetsos, 2015), RDU suggests that decision-makers evaluate the value of their options relative to not one, but two reference points: the minimum and maximum rewards available in any given scenario. In this view, what PT identifies as a reference-point could be nothing more than the product of a utility function that adapts to the distribution of possible rewards: the point at which a sigmoidal curve inflects from convex to concave (and a shape akin to a neuron's tuning curve; Carandini & Heeger, 2012; Webster, Werner, & Field, 2005).

Because studies on reference-dependence generally focus on identifying a unique reference-point (Baillon et al., 2015), or on describing behaviors under specific reference predictions ((Allen et al., 2016); Crawford & Meng, 2011; Wenner, 2015), there is, as of yet, no way of corroborating or contradicting the previous hypotheses on the emergence of reference-points. The few studies that consider shifts in preferences generally do so in the context of a specific reward distribution and on the timescale of laboratory choice experiment: that is, they document reference-point changes following the wins or losses of risky gambles ((Arkes et al., 2008, Arkes et al., 2010); Shi, Cui, Yao, & Li, 2015); never the impact that changes in expectation have on decision-making over days, weeks, or months. Concurrently, little is known about the impact of a task's structure on preferences, nor how different reward statistics might translate to different reference-points and utility functions.

This study addresses the above gap by investigating the formation of reference-dependent utility in our close relative the macaque monkeys. Macaques are evolutionarily comparable to humans, and our laboratory setting allowed for repeated testing with much larger trial numbers and longer periods of experimentation. Most importantly, since monkeys acquire knowledge about rewards via experienced outcomes rather than from verbal or written instructions, behavioral measurements exclude potential confounds from language and higher numerical ability. Thus, we investigated how the distribution of rewards experienced in a binary choice task - defined on different reward magnitudes and spreads - shaped the reward preferences of rhesus macaques (a species that displays many, if not most, of the fundamental choice patterns that humans display; Heilbronner and Hayden, 2013, Heilbronner and Hayden, 2016; Stauffer, Lak, Bossaerts, & Schultz, 2015).

We presented macaques with several sets of risky choice options in which the distribution of reward magnitudes remained stable for weeks at a time, then suddenly shifted to a new distribution (higher/lower magnitudes or wider/narrower spread). On each testing day, we fit the animals' choices with S-shaped utility functions that could explain both risk-seeking and risk averse choices (Genest, Stauffer, & Schultz, 2016; Stauffer, Lak, & Schultz, 2014). We then looked at how the animal's risk preferences changed as a function of the reward distribution they experienced. We found that, while utilities stayed relatively put for periods during which a single reward distribution was experienced, the animals consistently shifted their preferences when a novel reward distribution was introduced. Specifically, the shape of estimated utility functions reflected the lowest and highest rewards that the monkeys had experienced over the course of the preceding weeks – doing so even if those rewards were now not a possibility. From these findings, we suggest that - far from being fixed and abstract – the utility functions estimated from monkeys' choices reflect preferences that change and adapt given the knowledge that these animals accumulate over time. A functional ‘reference’ for our monkeys, therefore, likely captures the range of one's expectations rather than a singular, context-specific value.

## 2 Methods

### 2.1 Animals

Three male rhesus macaques (*Macaca mulatta*) weighing 11.2, 15.3, and 13.2 kg (Monkeys A, B, and C, respectively) participated in this experiment. All animals used in the study were born in captivity, at the Medical Research Council's Centre for Macaques (CFM) in the UK. The animals were pair-housed for most of the experiment; monkeys B and C shared an enclosure. The animals ranged in age from 5 to 8 years old, and all subjects had previous experience with the visual stimuli and experimental setup (Ferrari-Toniolo, Bujold, & Schultz, 2019).

This research has been ethically reviewed, approved, regulated, and supervised by the following institutions and individuals in the UK and at the University of Cambridge (UCam): the UK Home Office implementing the Animals (Scientific Procedures) Act 1986 with Amendment Regulations 2012 and represented by the local UK Home Office Inspector, the UK Animals in Science Committee, the UK National Centre for Replacement, Refinement, and Reduction of Animal Experiments (NC3Rs), the UCam Animal Welfare and Ethical Review Body (AWERB), the UCam Biomedical Service (UBS) Certificate Holder, the UCam Welfare Officer, the UCam Governance and Strategy Committee, the UCam Named Veterinary Surgeon (NVS), and the UCam Named Animal Care and Welfare Officer (NACWO).

### 2.2 Behavioral task and training

Rhesus macaques are the most commonplace species of non-human primates found in scientific research (Capitanio & Emborg, 2008). There is thus a rich literature reproducing human economic choices with them, the most relevant for us being that rhesus macaque choices can be successfully modeled using PT (Farashahi, Azab, Hayden, & Soltani, 2018; Ferrari-Toniolo et al., 2019; Genest et al., 2016; Stauffer et al., 2015). In addition, macaque experiments allow us to control the pre- and post-experimental environments in ways not possible for human studies – we can ensure that experimental variables are independent of rewards and choices made outside of the experiment (Chen, Lakshminarayanan, & Santos, 2006).

Each animal used a left-right joystick (Biotronix Workshop, University of Cambridge) to make choices between reward-predicting stimuli presented on a computer screen. After each choice, the animals received their chosen reward in the form of a specific blackcurrant juice quantity delivered probabilistically (matching the probabilities indicated by each stimulus). Importantly, although the animals were familiar with the blackcurrant juice used as a reward (which they sometimes received in their home cage in quantities of 20–50 ml), the environment, form, and quantity of reward delivery differed between the laboratory and the home cage to avoid generalization of reward quantities. In the former, experimental rewards were delivered in the experimental laboratory via a fixed spout in front of the mouth with well-controlled sub-milliliter quantities.

**Fig. 1:**
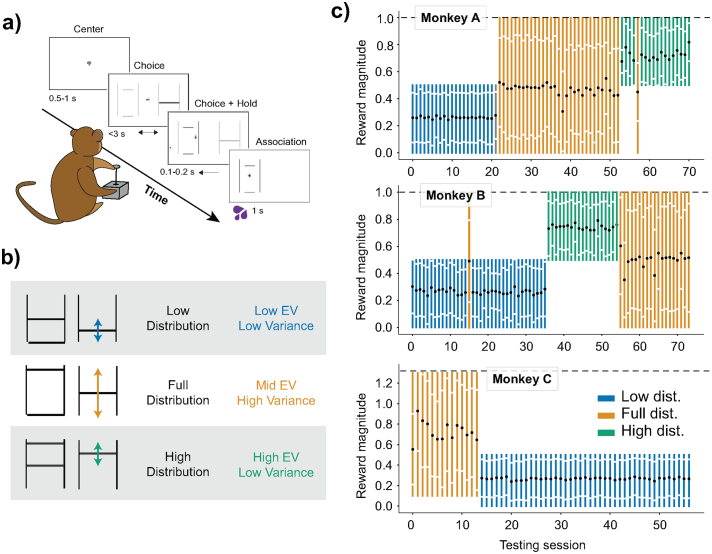
Experimental design and timescale. a) Binary choice task. The animals chose one of two gambles with a left-right motion joystick. They received the blackcurrant juice reward associated with the chosen stimuli after each trial: the reward's magnitude and probability of delivery were signaled by the vertical position and width of a horizontal line as set between two vertical ones. Times, in seconds, indicate the duration of each of the task's main events. b) Experimental reward distributions. Choices were made in one of three experimental reward distributions. In the low distribution, choice options had juice magnitudes set between 0 ml and 0.5 ml during preference elicitation sequences. The high distribution involved juice magnitudes set between 0.5 ml and 1.0 ml during preference elicitation sequences (unique to Monkey A and B). The full distribution was set between 0 ml and 1.0 ml for Monkeys A and B and set between 0.1 ml and 1.3 ml for Monkey C. c) Monkeys' experienced specific reward distributions for consecutive days. Vertical lines represent the daily experimental session, in their tested order; the height of these lines signals the reward distribution tested (blue, low distribution; yellow, full distribution; green high distribution). Black dots indicate the mean magnitude of all rewards experienced on the day, the white dots represent the standard deviation on the mean. (For interpretation of the references to colour in this figure legend, the reader is referred to the web version of this article.)

The animals were presented with a simple visual stimulus consisting of one or two horizontal lines positioned inside a frame of two vertical lines depicting reward options that varied both in magnitude (i.e. liquid quantities, ml) and in the probability of a reward being delivered. Reward magnitudes were represented by the vertical position of the horizontal lines on the screen, whereas reward probability was represented by the length of the horizontal lines inside the framing lines (Fig. 1a). Safe (riskless) options were represented by singular full-width horizontal lines that touched both sides of the frames, whereas gambles with multiple risky rewards were represented by multiple horizontal lines within the vertical frame.

The animals were trained to associate these two-dimensional visual stimuli with blackcurrant juice rewards over the course of >10,000 single-outcome, imperative trials. In these trials, a single reward option was presented on either the left or right side of the screen. To obtain the cued reward, the animals were required to select the side on which the reward was presented. After imperative training, where only one option was presented, all experimental data were gathered within a binary choice paradigm in which the animals chose one of two reward options presented simultaneously. One option was always a gamble; the other was always a safe, guaranteed reward. Every choice trial began with a white cross at the center of a black screen, followed by the appearance of a joystick cursor. To initiate a trial the animal had to move the joystick cursor to the center cross and hold it there for 0.5–1 s. After a successful central hold, two reward options appeared to the left and right of the central cross (Fig. 1a). The animal had 3 s to convey its decision by moving the joystick to the side of its choice and holding it there for 0.1 s to 0.2 s, after which time the unselected option would disappear. The selected option remained on the screen for 1 s after reward delivery to strengthen any stimulus-reward associations with visual feedback. A variable intertrial period of 1–2 s (blank screen) preceded the next trial. Errors were defined as trials with an unsuccessful central hold, trials in which the animal failed to hold the selected side for the time required, or trials in which the animal made no choices. These error trials resulted in a timeout of 6 s, after which time the trial was repeated.

Reward options were presented in pseudorandom alternation on the left and right sides of the computer screen to control for any side preference. Event times were sampled at 2 kHz and stored at 1 kHz on a Windows 7 computer running custom MATLAB software (The MathWorks, 2015a; Psychtoolbox version 3.0.11), and all further analyses were done using custom Python code (Python 3.7.3, Scipy 1.2.1, Oliphant, 2007). Over the course of 63, 43, and 57 sessions an average of 259 ± 154 (mean ± sd) trials, 317 ± 118 trials, and 131 ± 75 trials were collected for Monkeys A, B, and C, respectively. Crucially, animals received the reward they selected after each trial. This ensured that they experienced the rewards they selected with minimal and constant delay, and contrasts with human studies where only a randomly selected subset of trials are rewarded at the end of experimental sessions. Delivering rewards after every trial also allowed us to capture preferences that were contingent on experiences unique to the task - similar delivery methods and reward amounts were not experienced in the housing environment.

### 2.3 Measuring preferences for specific reward distributions

To examine the degree to which preferences are shaped by available rewards, binary choice data were collected from choices between reward options affixed to different reward distributions (Fig. 1b). Three reward distributions were defined in terms of their mean reward magnitude and the spread of possible options i) low-narrow distribution, where tested magnitudes were generally set between 0 ml and 0.5 ml; ii) high-narrow distribution, with magnitudes between 0.5 ml and 1.0 ml; and iii) full distribution, with magnitudes between 0 ml and 1.0 ml (0.1 to 1.3 ml for Monkey C). Importantly, every reward outcome (no matter which distribution) was repeated the same number of times for each session – thus, every reward was equiprobable (flat distribution). We set distributions and kept them fixed for multiple weeks, measuring the effects of reward distribution over weeks rather than across blocks of a single experimental session (Fig. 1c). Monkey A experienced a low distribution for 22 days (0 ml to 0.5 ml), a full distribution of rewards for 31 days (0 ml to 1.0 ml), and a high distribution of rewards for 17 days (0.5 ml to 1.0 ml). Monkey B experienced the low distribution for 33 days, then 19 days of high distribution, followed by 18 days of full distribution. Monkey C, quite uniquely, offered a dataset with a longer timescale. He experienced the full distribution of 0.1 ml to 1.3 ml of reward for 14 days then switched to a low distribution of 0 ml to 0.5 ml for 54 weeks. After this, his preferences were measured over 43 days.

Utility functions were estimated for each reward distribution by presenting individual animals with a series of choices between a safe reward (probability of reward, *p* = 1.0) and a binary, equiprobable gamble (each reward *p* = 0.5) from which Von Neumann–Morgenstern type utilities were estimated. For our choice paradigm (i.e. the unique choices and the order in which they were presented), we used the fractile-bisection procedure (Machina, 1987). This involves successively dividing the distribution of possible utilities into progressively smaller halves (or fractals) and estimating at each step the magnitude of safe reward at which choices were indifferent against the specific gamble being tested, as done in our laboratory before (Genest et al., 2016; Stauffer et al., 2014). The resulting magnitude is termed certainty equivalent (CE) and represents the subjective value of the safe reward that is equivalent to the value of the gamble.

**Fig. 2:**
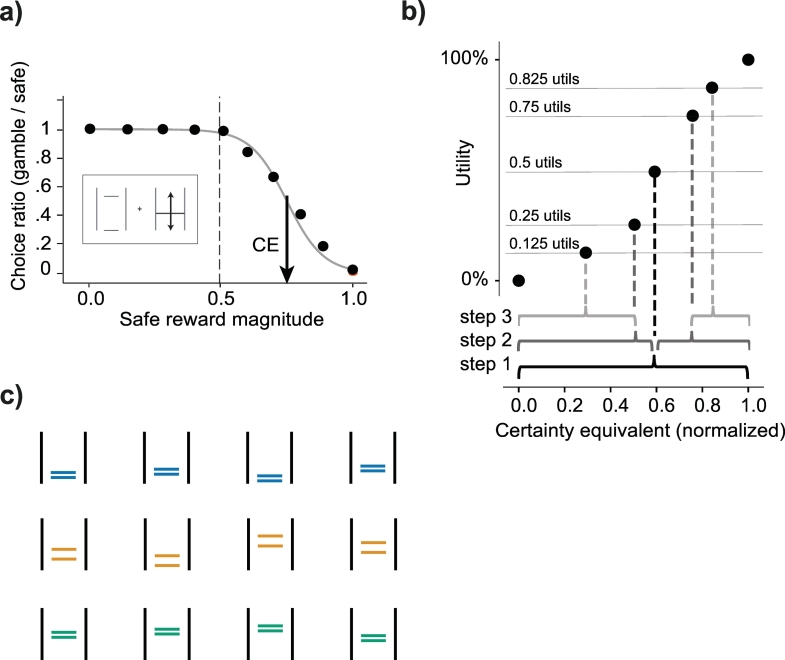
Estimating certainty equivalents and utility functions. a) Basic choice behavior and estimation of certainty equivalents. Animals chose between a safe reward and a gamble on each trial. The safe rewards alternated pseudorandom on every trial – never going above or below the highest and lowest magnitudes tested in the daily reward distribution. Each point is a measure of choice ratio: the animal's probability of choosing the gamble option over various safe rewards. We fit psychometric softmax functions (Eq. (1)) to these choice ratios, separately for each day, and recorded the certainty equivalent (CE) of individual gambles as the safe magnitude for which the probability of either choice would be 0.5 (black arrow). The dashed vertical line indicates the expected value (EV) of the gamble represented in the box. b) Estimation of utility using the stepwise, fractile method. In step 1, the animals were presented with an equivariant gamble comprised of the maximum and minimum magnitudes in the tested reward distribution. The CE of the gamble was estimated and assigned a utility of 50%. In step 2, two new equivariant gambles were defined from the CE elicited in step 1. The CEs of these gambles were elicited and assigned a utility of 25% and 75%. Two more gambles are defined in step 3, from the CEs elicited in step 2. Their CEs were then assigned a utility of 12.5% and 87.5%. Parametric utility functions, anchored at 0 and 1, were fitted on these utility estimates (see *methods*). c) Equivariant, equiprobable gambles presented in out-of-sample validation sequences. Sets of four gambles, unique to each reward distribution, were used to validate the risk attitudes predicted by the DCM-derived utilities. The CEs of these gambles were measured (see panel a) and the difference between CEs and the specific gambles' EVs signaled the animals' risk attitudes: if the difference was positive, the animals were risk-seeking, if the difference was negative, the animals were risk averse.

The first step of the procedure involved presenting the animals with choices between this gamble and varying safe rewards (in 0.05 ml increments); in these choices, the safe reward that was equivalent to the gamble in utility terms was identified (i.e. the safe reward chosen in equal proportion to the gamble; see Fig. 2a, b). To estimate this safe reward, the following logistic sigmoid curve was fitted to the proportion of safe choices versus gambles for each of the gamble/safe pairing:

(1) $$P_{\mathrm{ChooseSafe}}=1/\left(1+e^{-\left(\frac{{\mathrm{SafeReward}}_{\mathrm{ml}}-x_{0}}{\sigma }\right)}\right)$$

The probability of the animal choosing a safe reward over the 0.5 utility gamble (*P*_*ChooseSafe*_) was contingent on the safe option's magnitude (${\mathrm{SafeReward}}_{\mathrm{ml}})\;$and the two free parameters, x_0_: the x-axis position of the curve's inflection point, and σ: the function's ‘temperature’. Importantly, this function's inflection point represented the exact safe magnitude for which the animal should be indifferent between the set gamble and a given safe reward. Then we assigned utility to the lowest juice reward amount (0.0 utils) and highest juice amount (1.0 util) for the currently tested distribution (Fig. 2b). Since the animals only experienced trials set between these reward magnitudes, this constrained all utility estimates between 0 util and 1 util. The x_0_-parameter could thus be used as a direct estimate of the gamble's CE: at choice indifference, the safe reward had the same utility as the equiprobable gamble (*p* = 0.5 each outcome) formed of these two magnitudes, which amounted to 0.5 (i.e., [0.5 × 0.0 utils] + [0.5 × 1.0 util]). In the subsequent step, a new equiprobable gamble was set between 0 ml and the first CE's ml value. The CE elicitation procedure was then repeated (logistic fitting, Fig. 2a); their CE had a utility of 0.25 utils (1/4 of maximal utility). In the next step, two new equiprobable gambles were set between the first CE's ml value and the maximum magnitude of the currently tested reward distribution, i.e., 0.5 ml; their CE had a utility of 0.75 utils (3/4 of maximal utility). Crucially, gamble/safe pairings for both gambles were interwoven in the same sequence – to ensure a similar spread in the presented rewards. Only sequences that contained a minimum of three different choice pairs (repeated at least 4 times) were used in the elicitation of CEs, and only those fractile sequences where at least 3 utility values could reliably be estimated were used in further analyses. The CEs assigned to each utility level, in each reward distribution, were compared via two-way ANOVA.

### 2.4 Parametric estimation of utilities from aggregate and single choices

Since the fractile method relied on stepwise, chained measurements (where later metrics depend on earlier ones), utility functions were estimated using a discrete choice model (DCM) fit to individual trials. By fitting a model on individual choices rather than aggregate CE sequences, we avoided the propagation of estimation errors from earlier steps onto the next, and therefore reduced estimation biases for individual utility functions (Abdellaoui, 2000). Keeping the CE measures, however, allowed us to validate the parametric estimates of utility with the utility-CEs estimates from the fractile procedure.

We built our choice model as is commonly done (McFadden, 2001; Stott, 2006): by modeling the likelihood that monkeys would choose the left option over the right one, given a set noise level, a side bias, and the utilities associated with the left and right options. This was achieved using a logit function:

(2) $$P_{\mathrm{chooseLeft}}=\frac{1}{1+e^{-\lambda \left({\mathrm{EU}}_{\mathrm{Left}}-{\mathrm{EU}}_{\mathrm{Right}}-\theta \right)}}$$

where the probability of choosing the left option was a function of the expected utility difference between the left and right options, the temperature (or noise) parameter, *λ*, and *θ* which captured side bias parametrically. The expected utility of each option (*EU*_*Left*_, *EU*_*Right*_), as a function of their probability (p) and the utility function *U(m)*, was given by the functional form:

(3) $$\mathrm{EU}=p\times U\left(m\right)$$

Probability distortions are symmetric and usually minor at *p* = 0.5 (Ferrari-Toniolo et al., 2019; Stauffer et al., 2015); and so, to simplify our model, therefore we assessed the value of options solely as EU. The model's best-fitting parameters were estimated by minimizing the following cumulative log-likelihood function:

(4) $$-\mathrm{LL}\left(\theta |\;y\right)=-\left(\sum_{i=1}^{n}\;y_{i}\times \log\left(P_{\mathrm{Choose Gamble}}\right)+\sum_{i=1}^{n}\;y_{i}^{\prime }\times \log\left(P_{\mathrm{Choose Safe}}\right)\right)$$

where *y* and *y’* indicated a left or right choice (0 or 1), respectively, for each trial *i*; *n* was the total trial number for the session.

We compared several parametric utility functions, within the above model, to ensure the most reliable utility predictions; the best-fitting functions would then be used for all further analyses. In accordance with the assumptions of the fractile method described earlier, each of these functions was to be anchored at 0% to 100% on the y-axis –– and we normalized the rewards on which they were fit to be between 0 and 1. Carrying on this assumption allowed us to better compare the utilities later on.

We fit a 1-parameter power function (U_1-Power_), a common practice in economics,

(5) $$U_{1-\mathrm{power}}\left(m\right)=m^{\alpha }$$

with *m* for juice magnitude (in ml) of a given reward outcome and α as power parameter of the function (if α < 1 utility function is convex, if α > 1 utility function is concave).

Then, we fit two 2-parameter functions (U_2-Prelec_, U_2-SCDF_),

(6) $$U_{2-\mathrm{Prelec}}\left(m\right)=e^{-\beta \times {\left(-\ln\left(m\right)\right)}^{\alpha }}$$

with α-parameter as a curvature parameter of the function (generally, if α > 1 the function is S-shaped, if α < 1 the function is inverse S-shaped), and the β-parameter controls the elevation (or height) of the function. Although typically used to capture probability distortions, this monotonic function allowed us to capture risk-attitude reversals in a single, continuous function (Prelec, 1998). The function also behaves as a power law (i.e. like Eq. (5)) when the α parameter is set to unity – its axiomatic roots allow it to represent both a power function and a sigmoid-type one.

(7) U2−SCDFm=β×mκ1/α,for0≤m≤β1−1−β×1−m1−β1/α,forβ<m≤1

is the 2-parameter CDF of a two-sided power distribution. Its α-parameter controls the function's curvature (in the same way it controls the curvature of Eq. (5)) with α > 1 leading to a utility function that is S-shaped, and α < 1 to one that is inverse-S shaped). The β-parameter controls the x-axis position at which the function's curvature inverts, that is, its inflection point (this function is the functional form of the aforementioned RDU; Kontek & Lewandowski, 2018).

Finally, we fit one 3-parameter function (U_3-Power_)

(8) U3−powerm=m−γα,form≥γ−β×γ−mα,form<γ

serving as the s-shaped power function generally used to model PT (Stott, 2006). The inflection of the power functions is set at the *γ* parameter, and the function accounts for any loss aversion through its β-parameter accounts. Again, α controls the curvature of the function (if α > 1 utility function is S-shaped, if α < 1 utility function is inverse S-shaped).

Sets of daily Bayesian Information Criterions (BIC) were defined on the log-likelihoods of the models ( *BIC*_*LL*_ = (*k* ×  *ln* (*n*)) − (2 × *LogLikelihood*) ). We selected the best fitting function using a one-way Friedman test followed by pairwise Wilcoxon signed-rank tests (Bonferroni-Holm corrected) and compared the estimated parameters specific to each reward range using a one-way MANOVA.

Cross-validation of the negative log-likelihoods was also performed. Each daily fit went through a 10-fold cross-validation procedure, whereby parameters were fit to 90% of daily choices and used to validate the model's predictions on the remaining 10%. Sets of 10 negative log-likelihoods were averaged for each daily fit, providing one average -LL per day, per model, that would corroborate or contradict the BIC metrics described above. Once a model had been selected, we only proceeded with fits that fell below our classification of parametric outliers: that is, those that fell within 3× the standard deviation of the average log-value of all monkeys' utility parameters. We did so, using the data for all monkeys, as together they provided a less conservative and more realistic metric for what would be a ‘feasible’ utility function across all days for any monkey, and what would not.

### 2.5 Defining preference adaptation metrics

**Fig. 3:**
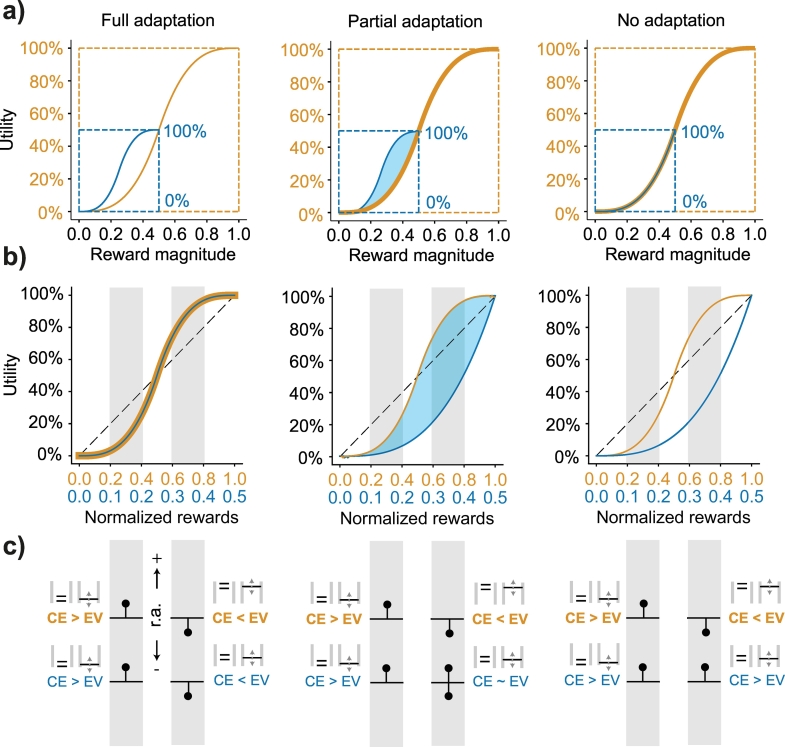
Schematic representation of full-, partial-, and non-adapting utilities estimated in low- and full-distributions of rewards. a) Scaled, identical utility functions in different reward distributions: the utility value of a 0.5 ml reward in the small distribution (blue curve, 100% utility) is scaled to the utility value of 0.5 ml reward in the large distribution (yellow curve). From left to right, utilities reshape assuming full-, partial-, and no adaptation. The three possibilities differ mostly in terms of the risk attitudes exhibited for rewards between 0 ml and 0.5 ml – under full adaptation they should differ, under no adaptation they should not. b) Utilities normalized according to the reward distribution from which they were estimated. Utilities are set on the same scale by normalizing across the domains of each function. Curves should overlap if utilities adapt fully (left) and fail to do so if there is no adaptation (right). If functions fail to adapt the low distribution utility is predicted to be identical to the first half of the full distribution utility curve. c) Predicting the direction of risk attitudes (r.a.) from utilities. For an equiprobable gamble made up of the two outcomes that fall at the edges of each grey shaded area, the horizontal black line depicts the expected value (EV) and the black dot above or below signals the direction in which we expect the certainty equivalent (CE). A black dot above the horizontal line signals risk-seeking behavior (or positive r.a.) and a CE of higher value than the EV, and a dot below the line signals risk averse behavior (negative r.a.). From left to right we again have predictions of r.a. given full-, partial-, or non-adaptive preferences. (For interpretation of the references to colour in this figure legend, the reader is referred to the web version of this article.)

We compared the utilities estimated from choices in different reward distributions in one of two ways: the first, assuming that preferences were fixed and did not adapt to the distribution of possible rewards in a task (Fig. 3a); the second, assuming that preferences fully adapted to the reward spread and magnitude of the task at hand (Fig. 3b). To test for the former, utilities estimated in narrow distributions (i.e., low- and high-distribution) were compared to the full-distribution ones. For the assumption of full adaptation, utilities were compared sequentially – looking for differences in the shape of the utilities between different distributions.

Most parametric utility functions had a unique inflection point, defined as a singular point where the utility function's curvature reversed, and where the function's first derivative was maximized. This inflection identified the precise reward magnitude for which the animals' risk attitude changed, and served as a good indicator for where and how the animals' preferences would change depending on the variance and mean of the reward distribution. The inflection points elicited in different distributions were compared using a Kruskal Wallis test with Bonferroni-Holm corrected post-hoc analysis (Wilcoxon test).

Another metric, the curvature ratio (CR) was defined as the normalized area under the utility functions (the function's area divided by the total area in each distribution). The CR provided a direct, normalized metric of the convexity/concavity interplay of daily utility estimates – reflecting overall risk attitude to a greater degree than inflection points. A linear utility function would have a CR of 0.5, as would perfectly symmetric S- or inverse S-shaped utilities. A CR above 0.5 indicated that the functions fell above the diagonal and predicted more risk averse choices; conversely, a CR under 0.5 reflected more risk-seeking choices. The CRs measured in the different distributions were also compared using a Kruskal Wallis test followed by pairwise Wilcoxon rank-sum comparisons (Bonferroni-Holm corrected).

A final series of metrics, defined as adaptation coefficients, allowed for the quantification of relative changes in CRs between utilities that had been estimated in consecutive reward distributions.

A sequential adaptation coefficient (SAC) was calculated as:

(9) $$\mathrm{SAC}=\frac{\left(\int_{\min}^{\max}U_{n}\left(m\right)\mathrm{dm}-\int_{\min}^{\max}U_{n-1}\left(m\right)\;\mathrm{dm}\;\right)}{\int_{\min}^{\max}U_{n-1}\left(m\right)\;\mathrm{dm}}$$

It captured changes in the median utility of a given reward distribution *n* (*U*_*n*_(*m*)), where *m* represented every reward between the minimum and maximum rewards in the tested distribution, relative to the median utility function in distribution *n-1* (*U*_*n*−1_(*m*)). Since all parametric functions were defined from 0 to 1, comparing the area under each curve gave us a direct measurement of the difference between the utilities that captured preferences in consecutive reward distributions.

A second coefficient, the general adaptation coefficient (GAC), compared the utility of low- and high-reward distributions to the utility estimated from an animal's full reward distribution. The GAC placed the narrow-distribution utilities (i.e., the low- and high-distribution ones) relative to the shape of the full-distributions utility function. That is, a GAC of 0 would indicate that the narrow-distribution utilities are but segments of a fixed full-distribution one, whereas a GAC of 1 suggested that utilities kept a similar form but shifted to represent fixed preference relationships that simply mapped onto a new distribution. For any GAC where 0 < GAC < 1, preferences would be said to have partially adapted. To calculate this, narrow distribution utilities were rescaled to map onto the full distribution ones: the maximum value of the low-distribution became the utility value of the full-distribution utility at 0.5 ml, and the utility value of the full-distribution utility at 0.5 ml became the minimum value of the high-distribution. Then, the median utility of the full distribution (U_Full_) was rescaled (into *U*_*adapt*_) to match the domain and image of narrow distribution utilities (U_Low−distribution_ and U_Hugh−distribution_). The GAC was given by

(10) $$\mathrm{GAC}=\frac{\left(\int_{\min}^{\max}U_{\mathrm{partial}}\left(m\right)\mathrm{dm}-\int_{\min}^{\max}U_{\mathrm{full}}\left(m\right)\;\mathrm{dm}\;\right)}{\left(\int_{\min}^{\max}U_{\mathrm{adapt}}\left(m\right)\;\mathrm{dm}-\int_{\min}^{\max}U_{\mathrm{full}}\left(m\right)\;\mathrm{dm}\right)}$$

where *min* and *max* are the minimum and maximum reward magnitudes in a narrow distribution condition. A GAC of 1 signaled full adaptation while a GAC of 0 indicated that no adaptation had taken place. Crucially, the GAC metric took no account of the order in which reward distributions were tested; it relied instead on the full-distribution utility functions as a comparison template. Both the SAC and GAC's accuracy in identifying “no-adaptation” were measured via Monte Carlo simulations that assumed monkeys' choices were guided by a singular non-adapting utility function.

### 2.6 Validating utility predictions from out-of-sample certainty equivalents

To validate the predictions of the utility functions, CE measures were also gathered from binary choices presented outside of the fractile sequences. In other words, the CE of gambles not used for utility estimation were gathered separately and used to corroborate the risk attitudes predicted by the DCM-derived utilities. Two of the three animals were presented with three sets of four gambles unique to each reward distribution for which we estimated CEs. We used these 12 CEs to validate the risk attitude predictions of the utility function estimated in each distribution. The gambles in the narrow reward distributions had a spread of 0.15 ml, while gambles in the full distribution had a spread of 0.30 ml – keeping the relative spreads equivalent across the distributions. Gamble means were also, once normalized, centered around the same relative values. In percentage points, each gamble spread over 30% of the reward distributions, and gamble was centered at a value representing 25%, 45%, 65%, or 85% of the reward distribution (Fig. 2c).

Taking the difference between the CEs of these gambles and their expected value (EV) as a proxy for risk attitude (CE – EV), the risk attitude estimated from these CEs were compared with the predictions from the discrete-choice utility curves. If the CE – EV metric were positive, it signaled that the animals were risk-seeking (Fig. 3c). If instead, the measures were negative, the animals could be seen as being risk averse. Because of this, if the utility models imposed an S-shape that was unrealistic (and a consequence of the function used) the CE – EV fits would expose it right away: they would not transition from risk-seekedness to risk-aversion. These measures were repositioned relative to the inflection point at which DCM-derived utilities predicted a reversal of risk attitudes (i.e., the point of risk neutrality. Linear regressions were fit to the repositioned CE – EV metrics to validate DCM-derived infections:

(11) $$\mathrm{CE}–\mathrm{EV}=\beta_{0}+\beta_{1}\left(\mathrm{EV}-\mathrm{inflection}\right)$$

In the model, *β*_0_ Indicated the point at which CE measures became risk-neutral, and *β*_1_ Paralleled the ‘depth’ of utility's curvature. These regressions allowed for the validation of predicted risk-attitudes.

## 3 Results

### 3.1 Experimental design

**Fig. 4:**
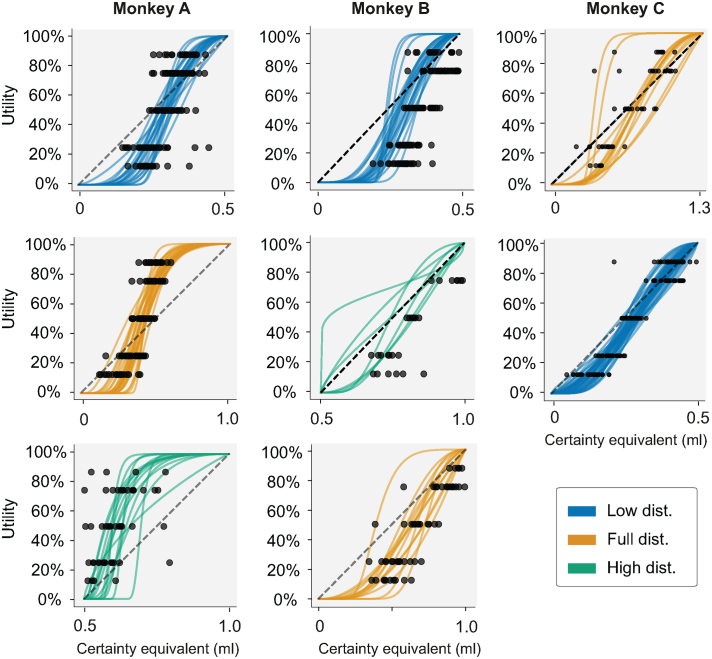
CEs and utility functions elicited from daily fitting procedures. Order of distributions tested is captured vertically. Black dots represent CE-utility pairings elicited in individual experimental sessions using the fractile method; colored lines are discrete-choice utilities fitted (*U*_2−*Prelec*_) to the individual decisions that led to daily fractile CEs (blue, low narrow distribution; yellow, full distribution; green, high narrow distribution). Utility fits for Monkey A, from top to bottom, represent 20 days (out of 21 due to outlier removal), 26 days, and 15 days. For Monkey B, we have 23 days, 7 days, and 13 days. Finally, Monkey C has a total of 13 days for the top panel, and 43 days for the lower one. In all cases, convexity of the functional fit signals risk-seeking behavior, concavity signals risk aversion. (For interpretation of the references to colour in this figure legend, the reader is referred to the web version of this article.)

To investigate the adaptation of utility functions to different reward distributions, macaque monkeys were presented with sequences of binary choices while reward distributions were kept constant over consecutive days, and then suddenly changed. Because of this, the animals experienced periods of relatively low reward magnitudes, periods of relatively high magnitudes, and periods with a mix of both (Figs. 1c, 4). On each day the animals were presented with either a utility estimation sequence, an equivariant gamble sequence (out-of-sample validation), or both.

In utility estimation sequences, utility measurements were derived from the choices that animals made between sets of gambles and safe rewards. Using the fractile method (see *Methods*), utilities were derived from the certainty equivalents (CEs) of specific sets of binary, equiprobable gambles (*p* = 0.5 each outcome; the magnitude of safe reward that was subjectively equivalent to the gamble). In validation sequences, the animals' risk preferences were measured directly using the CEs of out-of-sample binary, equiprobable gambles. These measurements were then used to corroborate the risk attitude predicted by utilities estimated in elicitation sequences.

Sets of daily utilities were estimated for each reward distribution that the monkeys experienced. The way reward magnitudes (CEs) mapped onto these utilities could then be compared within and between the different rewards distributions. To do so, and because utilities were defined from 0% to 100% regardless of their distribution, the CEs were normalized relative to the maximum and minimum magnitudes in the appropriate reward distribution (see, Fig. 4). This allowed us to compare the relative change in preference intensity across the different reward distributions' domains. As expected, higher utility values mapped onto higher reward magnitudes (higher CEs), but how they did so differed significantly depending on the current distribution. The same utility levels in different reward distributions (12.5%, 25%, 50%, 75%, and 87.5% of their respective 1 util) did not map onto the same relative magnitudes (i.e., normalized CEs). We confirmed this statistically using a two-way ANOVA with the main factors being the utility level tied to individual CEs, and the reward distribution from which they had originated. As one would predict, the ANOVA identified a significant main effect of utility level on the value of the estimated CEs (Monkey A: F(4, 292) = 64.222, *p* = 6.637 ×10^−39^; Monkey B, F(4,192) = 50.51, *p* = 4.107×10^−39^; Monkey C: F(4, 293) = 538.261, *p* = 1.258×10^−133^). That is, higher reward magnitudes were associated with higher utility values. The distribution in which utility-specific CEs had been estimated also had a significant main effect on the value of the estimated CEs (Monkey A: F(2, 292) = 349.918, *p* = 2.922×10^−78^; Monkey B, F(2,192) = 8.994, *p* = 0.003×10^−3^; Monkey C: F(1, 293) = 32.773, *p* = 2.563×10^−8^). Together, these corroborated what we could see graphically (Fig. 4): higher CEs correlated with higher utilities in all distributions, but these CEs were of relatively lower value once a shift from low- to full- or high-distribution had occurred. Supporting the two other main effects, we found a significant interaction effect of utility level and distribution on the estimated CEs, in two of the three animals (Monkey A: F(8,292) = 1.005, *p* = 0.432; Monkey B, F(8,192) = 5.217, *p* = 1.829×10^−5^; Monkey C: F(4, 293) = 8.593, *p* = 1.435 ×10^6^). This, we found, was due to changes in the steepness of the utility-CE pairings between the different reward distributions – rather than simply shifting and recalling, utilities in different distributions seemed to follow different patterns.

### 3.2 S-shaped utilities best fit choices

Because of the fractile method's reliance on aggregate, chained data to measure utilities (i.e., CEs; Farquhar, 1984; Machina, 1987), parametric utility functions were fit to daily individual choices using a discrete choice model (DCM; see Eqs. (2), (3), (4)). The daily utility functions fit to the data were then used to understand the relationship between the monkeys' risk attitude in each of the tested reward distribution.

Several different functional forms of utility were compared (Eqs. (4), (5), (6), (7), (8)); the most reliable function was then used in further analyses. Power functions are commonly used to model utility functions. We, therefore, fit a 1-parameter power (U_1-Power_), 2-parameter CDF of a two-sided power (U_2-SCDF_), and a 3-parameter anchored power functions (U_3-Power_) to the animal's CE-utility pairings. In addition to power-type functions, we looked at functions typically reserved for probability distortion modeling (Ferrari-Toniolo et al., 2019; Stott, 2006): the 1-parameter Tversky function (U_1-Tversky_) and the 2-parameter Prelec (U_2-Prelec_) – two monotonic functions that could readily take on the s-shape prescribed by PT, as well as mimic the shape of their simpler power function counterparts. All functions mapped reward magnitudes onto values from 0 to 1 (i.e., 0% to 100% of normalized utilities), and all but the 1-parameter power function could capture both risk-seeking and risk averse behaviors within the same reward distribution.

In line with the utility values derived from the fractile measurements, and because previous experiments with the same animals had identified negligible probability distortions for *p* = 0.5 (Stauffer et al., 2015), probabilities were treated as objective. That is, choices in the model relied on objective probabilities but subjective utilities. The parameters that best described individual choices in each model were estimated through maximizing the cumulative log-likelihoods of the DCMs defined on individual experimental sessions (Eq. (4); see methods).

**Fig. 5:**
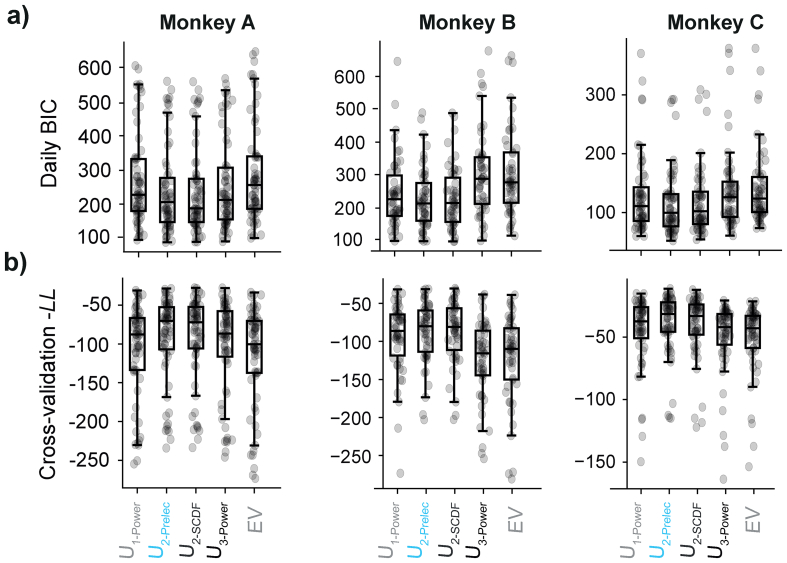
Model comparisons within and across fitting procedures. Various models (differing on their utility function) are contrasted, the 2-parameter Prelec model that was used throughout this study appears in blue (U_2-Prelec_). These fits were also compared to a model built solely on expected value (where utility is the objective reward amount). a) Model selection using Bayesian Information Criterion. Daily BIC scores were calculated for each utility function using the log-likelihoods estimates of each discrete choice models fit. Lower BIC scores indicated better fits between the discrete choice model (DCM) predictions and individual measured choices pairings. b) Model selection using Cross-Validated negative log-likelihood estimates. Each daily fit went through 10-fold cross-validation; each data point thus represents the average, cross-validated negative log likelihood of each day. Higher cross-validated -LL scores indicated better predictions of the discrete choice model (DCM) fit to 90% of daily choices relative the remaining 10% of choices. (For interpretation of the references to colour in this figure legend, the reader is referred to the web version of this article.)

To select the model (and, therefore, utility function) that best described the monkeys' choices, we used the Bayesian information criteria (BIC) from all fitted models; the model with the lowest median BIC represented the best fitting model. Of the five tested utility functions, the U_2-Prelec_ proved the most reliable in capturing choices (Fig. 5a). Though the model is normally reserved for probability distortion models, its flexibility resulted in the lowest BIC score as derived from the log-likelihoods of the discrete choice fits in 2 of 3 monkeys (Friedman test; Monkey A: F_r_(4, 310) = 219.091, *p* = 2.327×10^−45^; Monkey B: F_r_(4, 245) = 186.469, *p* = 2.221×10^−38^; Monkey C: F_r_(4, 275) = 177.122, *p* = 2.202 ×10^−36^). In Monkey A, the BIC of U_2-SCDF_ and the U_2-Prelec_ proved statistically indistinguishable. Cross-validation confirmed the BIC findings, the U_2-Prelec_ generated the highest average negative log-likelihoods across all functions (Fig. 5b). We, therefore, selected the U_2-Prelec_ model for all further analyses. Before proceeding, we identified any parametric outliers as above or below 3× the standard deviation of the average log-value of all monkeys' utility parameters. For that reason, we dropped 1 fit for monkey A (for all remaining utilities, see Fig. 4).

### 3.3 Risk preferences adapt to novel reward distributions

Each fitted utility function provided a pair of parameters that could be compared to those elicited in the same or different reward distributions. The curvature of these utility functions served as a direct indicator of the animal's risk attitude for any given magnitude. Convexity reflected risk-seeking behavior; concavity signaled risk aversion. From these parametric functions, three predictions could be made: utilities would either i) fully adapt to the novel reward distributions, ii) not adapt and remain constant (i.e., different parts of the same curve), or iii) utilities would partially adapt in a way that did not solely rely on the current reward distribution. To test for these predictions, further analyses were split into two sets of hypotheses. One set looked at utilities under the assumption that no adaptation had occurred, the other assumed full utility adaptation between each of the reward distributions. In the case of the no-adaptation assumption, the predictions from utilities on identical reward magnitudes in the narrow distribution and full distribution were compared (Fig. 3a). For the full adaptation assumption, the utilities from sequential reward distributions were normalized and compared, looking at any differences with the previous distribution's pattern of risk attitude (Fig. 3b, c). If neither assumption proved accurate, then the assumption would be that neither full nor no adaptation had taken place – that is, preferences would have partially adapted.

Comparing the functional parameters elicited in the different reward distributions provided us with a stringent test regarding the full adaptation assumption. In the U_2-Prelec_ function, the α-parameter represented the curvature of the function, while the β-parameter captured the relative height of the curve. If these were identical across conditions, similar patterns of utility reflected preferences regardless of unique reward magnitudes in the different reward distributions.

**Fig. 6:**
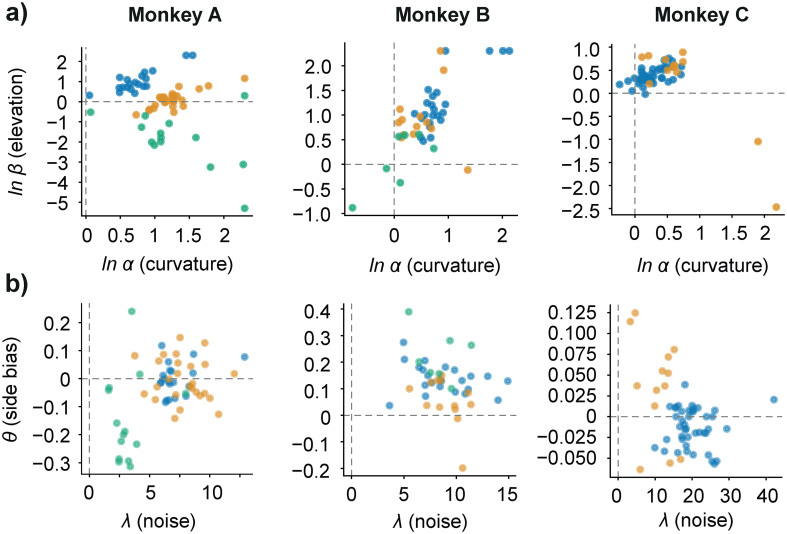
Daily parameter estimates from discrete choice model (DCM) fits. Parameter estimates from low-distribution utilities are in blue, full-distributions are in yellow, and high-distribution are green. a) Daily ln-transformed parameter estimates for the utility function. Each point represents a parametric utility fit on a daily set of choices. The x-axis captures the curvature parameter of the U_2-Prelec_ function: if **ln**α > 0 the function is S-shaped, if **ln**α < 0 the function is inverse S-shaped, it behaves as a power law if **ln**α ***=* 0**. The y-axis is elevation, or height: that is, the position of the function's potential inflection relative the diagonal. The higher its value, the higher in its domain the function's inflection can be found. b) Daily parameter estimates for the discrete choice logit function. Each point represents a parametric fit of the logit function that predicts the monkey's likelihood of choosing the left-hand reward option. The x-axis captures the noise parameter, i.e., the certainty with which they pick an option (lower values lead to more stochastic choices). The y-axis captures any side-bias that may govern choices; positive values imply a right-hand side bias, negative values imply a left-hand side one. (For interpretation of the references to colour in this figure legend, the reader is referred to the web version of this article.)

One-way MANOVA analysis on daily parameter estimates of the full model identified significant differences for all monkeys (Monkey A: F(2,58) = 30.806, Wilks's λ =0.309, *p* = 1.818×10^−13^; Monkey B: F(2,41) = 4.057, Wilks's λ = 0.701, *p* = 0.008; Monkey C: F(1, 54) = 17.655, Wilks's λ = 0.419, *p* = 3.752×10^−9^). There was a significant effect of reward distribution on the parameters elicited in each condition, for all animals (Fig. 6). Looking specifically at the ln-transformed parameters of the utility function (Fig. 6a), we found that there was a significant difference between parameters estimated in different reward distributions for all monkeys' α-, or curvature-, parameters (Monkey A: F(2,58) = 16.511, *p* = 1.473 ×10^−4^; Monkey B: F(2,54) = 6.414, *p* = 0.015; Monkey C: F(1,54) = 17.702, *p* = 9.808×10^−5^). We did, however, only identify significant differences between Monkey A's *β*, or elevation, parameters (Monkey A: F(2,58) = 99.564, *p* = 3.365 ×10^−14^; Monkey B: F(2,41) = 2.947, *p* = 0.094; Monkey C: F(1,54) = 0.652, *p* = 0.423). The noise (*λ*) and side bias (*θ*) parameters, variables also accounted for by the model (Fig. 6b), proved significantly different across distributions for monkeys A (noise: F(2,58) = 22.390, *p* = 1.470 ×10^−5^; side base: F(2,58) = 14.582, *p* = 3.288 ×10^−4^) and C (noise: F(1,54) = 34.112, *p* = 3.067 ×10^−7^; side base: F(1,54) = 17.843, *p* = 9.277 ×10^−5^). For monkey B, we only identified a significant difference between the side bias parameters (noise: F(2,41) = 0.021, *p* = 0.887; side base: F(2,41) = 5.655, *p* = 0.022).

To explore how these parametric differences influenced utility patterns in a way that was directly comparable between conditions, we compared the position of each utility function's inflection points – the reward magnitude at which the behavior predicted by the utility function flipped from risk-seeking to risk averse (or risk averse to risk-seeking, depending on the curvature of the utility function). The inflection crudely summarized choice predictions with a single metric – one that had been previously used to signal animals' ‘reference-points’ (Chen et al., 2006; Lakshminarayanan, Chen, & Santos, 2011). Importantly, since this metric was tied to CE values; one could easily observe if inflection points fell on similar magnitudes depending on the distribution in which it had been measured (Fig. 3a).

**Fig. 7:**
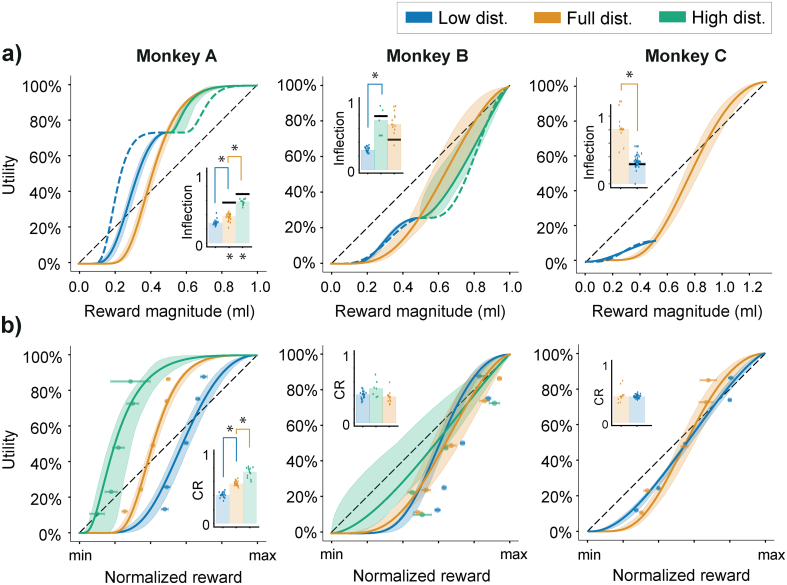
Discrete choice utilities reflect partial adaption to reward distributions. a) Scaled utilities estimated from discrete choice models (DCM). Each curve represents the median of daily, distribution-specific parameter estimates; 95% Confidence intervals were estimated via boostrapping said parameters (random sampling with replacement, *n* = 10,000). Dotted blue lines represent predictions full-distribution utilities predicted to fully-adapt to low-distributions. The dotted green lines represent similar full-adaptation predictions in the high distribution. Bar graphs represent the median inflection point, i.e., the reward magntiude at which the curve goes from convex to concave – points are daily inflection points. Upper asterisks (*) indicate differences between daily inflection estimates in two sequential distributions (Wilcoxon rank sum test); Lower asterisks (*) indicate significant difference between the median predicted inflection from the previous tested distribution and the true inflection estimates of the next distribution (Wilcoxon rank sum). b) Normalized utilities estimated from DCMs. Each curve is the median of daily, distribution-specific parameter estimates normalized according to the minimum and maximum rewards in the tested distribution. Again, 95% confidence intervals were estimated via boostrapping (random sampling with replacement, n = 10,000). Points represent mean normalized certainty equivalents ***±*** SEMs for each of the tested distribution. Bar graphs represent median curvature ratios (CRs) for each distribution; the relative concavity of each utility (concave >0.5; convex <0.5) – individual points are daily CRs. Upper asterisks (*) indicate significant differences between CRs estimated in sequential distributions (Wilcoxon rank sum). For each panel, blue comes from low-distribution utilities, yellow from full-distribution, and green from high-distribution. (For interpretation of the references to colour in this figure legend, the reader is referred to the web version of this article.)

From these inflection points, the assumption of no adaptation was tested by comparing the inflections gathered from the different reward distributions. We found significant differences between the distribution-specific inflections for all monkeys (Kruskal Wallis test; Monkey A: H(2,57) = 43.159, *p* = 4.247 × 10^−10^; Monkey B: H(2,40) = 31.103, *p* = 1.762 × 10^−7^; Monkey C: H(1,54) = 28.176, *p* = 1.108 × 10^−7^). This translated into significant pairwise differences (Wilcoxon rank-sum) for all but Monkey B's high and full distribution inflection points (Fig. 7a). Besides these, and for all other monkeys, the inflection points fell on different reward magnitudes for each of the reward distributions. If preferences had truly been non-adaptive, no significant difference across any of the conditions would have been observed.

Since none of the results cleanly corroborated the no-adaptation hypothesis, the next step was to test for full adaptation. Rather than comparing the absolute position of the utilities' inflection points, testing for full adaptation required predicting where inflection points from a past distribution would map onto the next distribution: the assumption being that if the same utility function simply shifted to a new distribution (i.e., fully adapted), the relative position of the inflection should be the same. An inflection at 0.3 ml in the low distribution, for example, would be placed at 0.15 ml in the full distribution, and vice versa. However, since an inflection of 0.3 in the low distribution would result in a negative magnitude when compared with the high distribution, inflections of lower value than the minimum reward were set at the minimum, and inflections of higher value than the maximum reward were set to the maximum. Wilcoxon rank-sum tests identified significant differences between all consecutive comparisons in Monkeys A (Z(45)_low-full_: −5.761, *p* = 8.351 × 10^−9^; Z(40)_full-high_: −4.790, *p* = 1.661 × 10^−6^), but none that reached significance for Monkey B (Z(29)_low-high_ = 1.103, *p* = 0.270; Z(20)_high-full_ = 1.941, *p* = 0.052) or C (Z(55)_full-low_ = 1.931, *p* = 0.053; Fig. 6a). From a full adaptation perspective, this suggested that, while Monkeys A and C had not fully shifted their reference to accommodate the new distributions, Monkey B's preferences seemed to follow the same relative pattern across all rewards distributions (corroborated the lack of significant difference between the utility functions' parameters).

From the inflection points, the picture that emerged was one of (at least) partial adaptation. That is, the significant differences between the inflection points corroborated neither the idea of fully- or non-adaptative preferences. Nevertheless, because inflection points carried no information about the risk attitude that followed or preceded them, the inflection points could be similar even if the animals' choices were not. To counter this, the previous comparisons were repeated using the area under each utility curve – a direct indicator of the convexity/concavity patterns within single utilities. Rather than representing a single point, the area under each curve reflected the order and intensity of risk-seeking or risk averse behavior throughout the reward distribution. Hereafter defined as curvature ratios (CRs, see methods), the areas calculated in each distribution were compared through Kruskal Wallis test (followed by pairwise Wilcoxon rank-sum post-hoc tests). The results validated the earlier findings from the inflection comparisons: sequentially, there were significant differences across distributions for Monkey A and B (Monkey A: H(2,58) = 51.253, *p* = 1.224 × 10^−11^; Monkey B: H(2,40) = 7.470, *p* = 0.024), but none of the post-hoc pairwise comparisons for monkey B reached significance once corrected for multiple comparisons (Wilcoxon rank-sum; Fig. 6b). This meant that Monkey B's preferences were much closer to being fully adaptive than not. There were no statistical differences between monkey C's CRs across conditions (Fig. 6b; H(1,54) = 0.042, *p* = 0.839). In essence, while the risk attitudes that Monkeys A and B exhibited differed between reward distributions, Monkey C seemed to exhibit relatively similar behavior in the two distributions (albeit with a slightly different inflection).

Taken together, these results suggest that while no animal (except perhaps Monkey B) demonstrated full adaptation, some form of partial adaptation had occurred across every distribution in every animal. More specifically, while not fully adapted, Monkey A and C's utilities did shift following changes in the task's reward statistics. Their inflection points moved, but not to the degree predicted by a full shift of the previous distribution's inflections. Where the two animals differed, however, was in the fact that Monkey C had maintained a very similar CR across conditions – likely due to the time elapsed between the different tests. Monkey B, on the other hand, maintained the relative inflection predicted across conditions and a similar utility shape.

### 3.4 Partial adaptation to reward distribution shapes risk preferences

Two final metrics served to quantify the degree to which each animal's DCM-utilities had adapted between the different reward distributions: a sequential adaptation coefficient (or SAC; Eq. (9)) and a general adaptation coefficient (GAC; Eq. (10)). The SAC served to quantify how the utilities adapted sequentially as a function of the preceding reward distribution, the GAC served to position utilities elicited in distributions with low and high means relative to adaptive or absolute utilities elicited from the full distribution.

The SAC represents the percent change in the CRs (the normalized areas under each curve) of successive utilities. It can be used to quantify differences in utilities within a single distribution, or, in this case, between the median utilities of different distributions. Importantly, the SAC allowed us to quantify utility adaptation on a normalized scale: if utility patterns were fully adapting (i.e., fixed shape regardless of the distribution), the SAC would gravitate to 0. On the other hand, the SAC would become negative if utilities became more convex (since the area under the utilities would become smaller), and more positive if utilities became more concave. The other coefficient, the GAC, compared the utility of the low- and high-distributions with the full reward distribution's utility function. Using the full-distribution utility as the ‘default’ utility shape, the GAC measured how different narrow utilities were – ranging from no or 0% adaptation (i.e., narrow utilities were but segments of an absolute full-distribution utility) to 100% adaptation (the utilities had a fixed form that simply adapted to new distributions). We used DCM-derived utilities to calculate these adaptation coefficients.

Using the SAC to quantify how median utilities changed between distributions, we found that the differences between utilities of Monkey A amounted to SACs of 0.37 and 0.34 for the full- and high-distributions, respectively; 0.11 and − 0.14 for Monkey B's high- and full-distribution, and 0.01 for Monkey C's low distribution. In utility terms, this meant that Monkey A's utilities predicted behavior that was 37% and 34% more risk averse in consecutive distributions. Monkey B also became more risk averse when going from the low distribution to the high distribution but became more risk-seeking again once choosing in the full distribution. The direction of these changes seemed to reflect the ‘position’ of the tested distributions relative to the past distributions the animals had experienced. In line with this idea, Monkey C had no recent experience with the full-distribution when low-distribution utilities were estimated; the measured utilities were thus almost identical.

The GACs calculated for each animal were also highly informative in positioning low- and high-distribution utilities relative to the full distribution ones (see dotted lines in Fig. 7a). Monkey A, for example, had a GAC of 0.51 for the small distribution, and a GAC of 0.21 for the high distribution. The high GAC essentially meant that the low-distribution utility was halfway between being only a segment of a fixed full-distribution utility and being a fully rescaled versions of the full-distribution utility; the low GAC suggested that high-distribution utilities were much closer to being segments of a larger, absolute utility function. For Monkey B, low-distribution utilities matched a GAC of 1.14, i.e. The utilities of the low distribution had an almost identical shape to those in the full-distribution, and the high-distribution utilities had a GAC of 0.69, a bit more than halfway between no- and full- adaptation. Monkey C, corroborating earlier findings, had a GAC between low and full-distributions of 0.98 – they were, for all intents and purposes, identical.

To ensure the reliability of the above findings, and of our DCM-procedure in correctly describing “no-to-full” utility adaptation patterns, we simulated and fit daily choice sequences that would mimic a no adaptation condition - using the same choice sequences the monkeys had previously experienced. Taking the median parameters of monkeys' full-distribution utilities, we first simulated utility-equivalent choices for low- and high-distribution fractile sequences, then estimated the parameters of distribution-specific utility functions given our DCM-fitting. Specifically, using monkey B's daily choice sequences, the above Monte Carlo simulation was run on each of the low- and high- distribution daily trial sequences (*n* = 1000), the daily median parameters were then collected to repeat SAC and GAC analyses. The procedure confirmed that - even in the extreme case of monkeys having non-adaptive utilities - the U_2-Prelec_ model would reliably identify parameters that best captured underlying preferences (and that, using the same number and type of trials that were used for our results; see Fig. S1). For example, given our estimation procedure, monkey B's theoretical low-distribution GAC of 0 translated to a simulated GAC of 0.01, and that of the high-distribution into a GAC of 0.10. We could thus be confident in the model's ability to capture and describe “non-to-fully” adaptive preferences.

Finally, going back to the original idea that preferences are shaped by one's expectations, we looked at the shape of each DCM-utility relative to the task's daily reward statistics. Though even the initial distribution's utility inflections never truly followed the task's mean reward (one-sample *t*-test; Monkey A: t(20)_low-distribution_ = 3.849, *p* = 0.001; Monkey B: t(23)_low-distribution_ = 2.534, *p* = 0.019; Monkey C: t(13)_full-distribution_ = 4.267, *p* = 1.103 × 10^−4^), the difference between mean rewards and inflections became markedly larger for Monkeys A and B when they were introduced to new reward distributions (Kruskal-Wallis test; Monkey A: H(2,58) = 39.218, *p* = 3.047 × 10^−9^; Monkey B: H(2,40) = `16.806, *p* = 2.242 × 10^−4^). Importantly, the differences were always skewed towards past distributions. As reward distributions changed, Monkey A and B's references appeared to lag in fully adapting to the new distributions. Monkey C, on the other hand, saw no differences between its two reward distributions (H(1,54) = 0.021, *p* = 0.884) – presumably because of the 54-week gap between the two sets of measurements.

### 3.5 Confirming risk preference reversals

While the DCM-fits largely predicted clear inflection points in monkeys' risk preferences (Fig. 7a), a number of inflection points near the maxima and minima of reward distributions (particularly in Monkey B) highlighted the need to validate the s-shaped utility pattern identified by the U_2-Prelec_ model.

To address this concern, we compared the risk attitudes predicted by the DCM-derived utilities to real risk attitudes measured in different, out-of-sample choices (i.e., validation sequences). The CEs of equiprobable and equivariant gambles were recorded in each of the reward distributions, and the differences between these CEs and the gambles' EVs (CE – EV) were used to indicate the animals' risk attitudes. Every gamble had a magnitude spread equivalent to 30% of the respective reward distribution, and their EV were anchored at 25%, 45%, 65%, and 85% of the testing distribution's magnitudes (Fig. 2c). If the difference between a gamble's CE and its EV (CE - EV) was positive, it reflected a risk-seeking attitude towards the gamble; if, on the other hand, this value was negative, the animal was said to be risk averse. These ‘validation’ measurements were gathered in two of our three animals (namely Monkeys A and B).

**Fig. 8:**
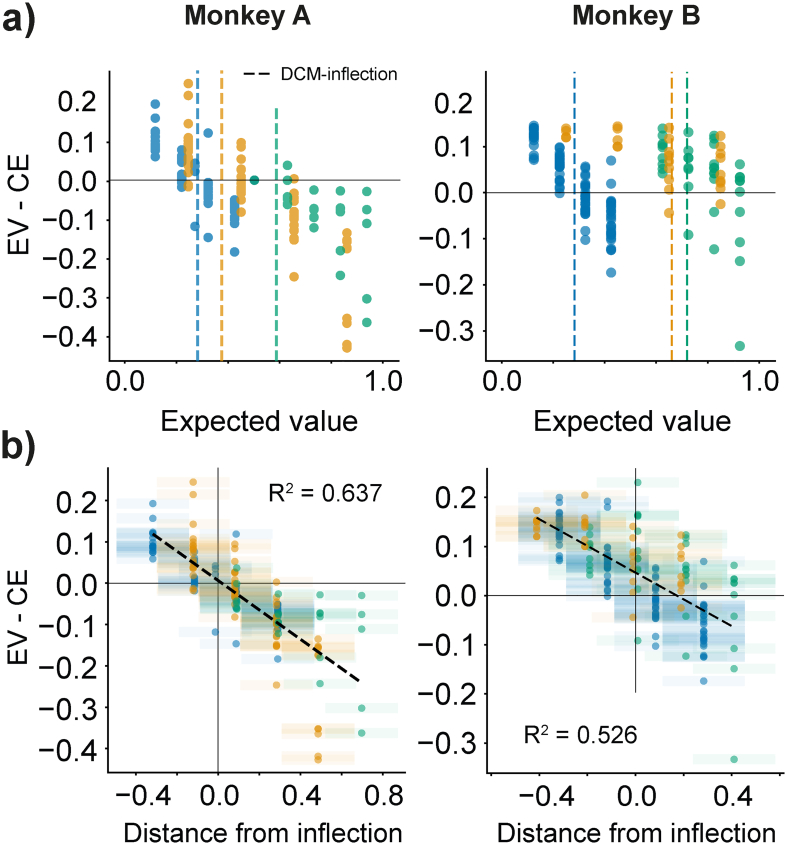
Discrete choice utilities better predict out-of-sample risk attitudes. a) Differences between the certainty equivalent (CE) and expected value (EV) of out-of-sample, equivariant gambles reflects the risk attitudes predicted by utilities. Each point represents a CE – EV measure from individual CE estimates. For CE-EV measures above 0 reflect risk-seeking behavior, points below 0 reflect risk averse behavior. The transition from risk-seeking to risk averse behavior should correlate with the inflection points predicted from utility functions: dotted lines represent the median inflection from discrete choice model (DCM)-derived utilities. b) DCM derived inflections (better) predict risk attitudes as measured in out-of-sample gambles. CE – EV metrics positioned as a function of a gamble's EV position relative the median DCM-derived inflection for each distribution. The x-axis captures the relative difference between the distribution's inflection point (in ml) and a gamble's EV (in ml). Dotted lines represent linear regression lines across all CE – EV measurements (Monkey A: *p* = 1.03 × 10^−35^; Monkey B: *p* = 1.90 × 10^−31^).

The CE - EV attitude predictions were compared to the risk attitude predictions from the DCM utility estimates (Eq. (11)). If the S-shaped pattern of utilities elicited for each animal were accurate, choices involving magnitudes that fell below the utility's inflection point should favor risky options whereas choices above it should favor safer options. We found that this was indeed the case and that CEs in all distributions reflected both the risk-seeking and risk averse choices predicted by DCM-derived utility functions in all but monkey B's full distribution (Fig. 8).

## 4 Discussion

The present study investigated the role of task-specific expectations in shaping the preferences of macaque monkeys. In line with human research on reference-dependent preferences (Arkes et al., 2008, Arkes et al., 2010; Koszegi & Rabin, 2007), the animals' risk preferences shifted following changes to the reward distribution they could expect from the task at hand. As the rewards that the task delivered got higher, the reward magnitude at which their risk attitudes shifted also became higher. Modeling the utility functions that best captured the animals' behavior, we found that changes in their risk-preferences mimicked the changes predicted in models like Prospect Theory (Kahneman & Tversky, 1979): the points at which utility shifted from convex to concave (i.e., when preferences shifted from risk-seeking to risk averse) closely followed what could be considered plausible expectations in the task.

Taking the position of S-shaped utilities as a proxy for the animal's expectations, our findings suggest that the monkeys partially adapted their preferences to account for new reward distribution in a task. While they readily adapted to novel rewards, they did not readily ignore (or forget) reward information that was no longer relevant to the task. Rather than relying solely on the current installment of the task to build their expectation, the monkeys appeared to also consider the distribution of past rewards – particularly the maxima and minima in a distribution - in shaping their preferences (i.e., their utility curve). The effect of range extremes has also been found to influence risk-attitudes in humans, and even pigeons (Ludvig et al., 2014). Interestingly, though macaque monkeys usually tend to base context-specific judgments on only a few trials (that is, they adjust their choices according to the outcomes of recent own choices; e.g., Barraclough, Conroy, & Lee, 2004; Grabenhorst et al., 2019; McCoy & Platt, 2005), our results suggest that experiencing new extremes may lead to slower but more entrenched changes for monkeys' preferences. Indeed, much like humans who can quickly adapt trial-by-trial (Hayden & Platt, 2009), our monkeys also showed that this likely takes place within much broader, more generalized learning. In these cases, while more reward is better than less, more is even better when there was less reward just before. Thus, our results are compatible with economic theory and experimental findings and provide an experimental approach for formalizing utility adaptation.

Monkeys A and B, for example, reliably shifted their reference point when possible rewards went from lower to higher magnitudes. When looking at the utility function that best represented their preferences, the animals' utilities appeared to scale instantly to represent the now broader realm of possible rewards. Conversely, when possible rewards were restricted to high magnitudes only (i.e. high-distribution), the animals did not adjust their preferences in a way that accounted for the unavailability of lower magnitudes – even after many days. Where they had previously been flexible in rescaling preferences, the animals' preferences in the high distribution (where low rewards were never delivered) stubbornly reflected the higher-half of full-distribution utilities. And while the shift from low to high distribution seemed to induce partial, almost full adaptation – the shift from full to high distribution reflected a move along a fixed, absolute utility instead.

The unusual testing regimen, i.e., the multi-week requirement to meaningfully quantify ‘long-term’ adaptation, meant that we could only commit a limited amount of days to record choices in each distribution setting. Because of the timeline, if a monkey would not participate in the experiment on a given day, we would lose a day of gathering results (they had observed the range, so we could not discount learning). For that reason, not all monkeys have the same amount of days recorded in each reward range. Future work on long-term adaptation may seek to address this limitation or allow for a more flexible timescale. With that said, the realities of our setting also provided an advantage: 54 weeks after our initial round of experiments, we were able to again work with Monkey C who had been allocated to a different experiment in the meantime. In doing so (and because he had since only experienced rewards between 0 and 0.5 ml), we were able to observe his preferences having readjusted to lower expectations. It allowed us to rigorously observe the adaptation of preference after over a year. While Monkeys A and B experienced every distribution in the span of just a couple of months, the effects of past high rewards on Monkey C would have been minimal. In that respect, it came as no surprise that Monkey C's lower distribution utilities took the form of fully rescaled full-distribution ones. A similar effect was seen in previous estimations with Monkey A's utilities (see, Genest et al., 2016).

The idea that preferences adapt to fit a given distribution is neither new nor unfounded (Brunswik, 1956; Gigerenzer, Hoffrage, & Kleinbolting, 1991; Glöckner, Hilbig, & Jekel, 2014; Weber & Johnson, 2008). Indeed, while prospect theory rests on reference-dependence, several newer models mimic RDU in that they claim that the values with which we imbue our options rely on the other options we have at our disposal (Hunter & Gershman, 2018; Loomes & Sugden, 2006; Parducci, 2012; Steward, Chater, Stott, & Reimers, 2003; Yaari, 1987). Likewise, it has long been known in psychology and neuroscience that distribution-adaptation is an inherent feature of the brain (Louie & De Martino, 2013). In sensory systems, for example, neuron's maximize their efficiency by tuning their firing rates to match the distribution of sensory signals (Carandini & Heeger, 2012; Laughlin, 1981) – the same is thought to occur, to varying degrees, in the brain areas that encode value (Burke, Baddeley, Tobler, & Schultz, 2016; Kobayashi, Pinto de Carvalho, & Schultz, 2010; Louie, Glimcher, & Webb, 2015; Padoa-Schioppa, 2009; Tobler, Fiorillo, & Schultz, 2005; Tremblay & Schultz, 1999). Specifically, and supporting the idea of distribution-dependent utility, neurons in the primate prefrontal cortex have recently been recorded adapting their firing rate to different reward distributions in a way similar to our animals' utility curves. In a study by Conen & Padoa-Schioppa, 2019, rhesus macaques only partially rescaled the value of juice rewards relative to the other possibilities in a given block of choices. When recording from neurons in monkeys' orbitofrontal cortex, the researchers found that the neural code mimicked behavioral measurements in that it partially adapted to match the specific reward distributions of different blocks within the broader context of all past rewards. Crucially, two processes seemed to drive this adaptation: the first, a slow and adaptive learning process about the outcomes one can expect (e.g., reinforcement learning; Bavard et al., 2018; Rudebeck & Murray, 2014; Wilson, Takahashi, Schoenbaum, & Niv, 2014), which involves the orbitofrontal cortex and its interaction with the dopaminergic system (for review, see Soltani & Izquierdo, 2019) and might explain the role of experience in shaping current preferences. The second process involves a rapid weighing of rewards relative to the decision-maker's present context (e.g., the canonical process of divisive normalization, whereby neurons tune their firing rates to match the distribution of available stimuli; Louie et al., 2013; Hiroshi Yamada, Louie, Tymula, & Glimcher, 2018; Zimmermann, Glimcher, & Louie, 2018). Building on the above, an interesting avenue that was not explored in the present study would be to quantify how rapid, within-session adaptation interacts with multi-day learning and expectation-building. The fractile method we used proves a limitation on this front since elicitation sequences require multiple ‘bisection’ blocks that do not cover the entire reward range. However, shorter, block-wise utility elicitation paired with the recent developments of ‘utility’ models that capture both learning and rapid contextual adaptation may prove useful for future work (e.g., Tymula & Glimcher, 2019; Webb, Glimcher, & Louie, 2020).

Partial adaptation is likely to underlie the brain's ability to maximize ‘local’ decisions, all while placing these decisions in a much broader context (i.e. relative past experiences; Conen & Padoa-Schioppa, 2019; Fairhall et al., 2001; Rustichini, Conen, Cai, & Padoa-Schioppa, 2017). When comparing similarly-priced wines, for example, we manage to select our favorite from relatively narrow distributions (similar prices) while still placing our selection relative to a much broader price distribution (our past experiences with wines). It has recently been suggested that this ability to flexibly optimize ‘local’ decisions while keeping track of past outcomes underlies the formation of cause-and-effect relationships in our thinking (Bavard et al., 2018). If this is the case, then the changes observed in our animals' utility functions point to the animals building complex expectations, or an internal model, about the rewards they could receive in the task at hand.

Overall, and in line with the current view from neuroeconomics, this study showed that the preferences of macaque monkeys' scale in a way that reflects both inherent properties (and indeed limitations) of the brain and the statistics of the environment at hand. It is intuitive to assume that the choices of monkeys, as well as those of humans as evolutionary close cousins, reflect a number of external influences, the most plausible being recent experiences or predictions of future variations. In this way, primates can tailor their choices to the best possibilities and thus maximize their returns.

## Author contributions

PMB, SF-T and WS designed the study, PMB and SF-T performed the experiments, PMB analyzed the data, PMB wrote the paper with comments by SF-T and WS.

## Declaration of Competing Interest

The authors declare no competing financial interests.

## Appendix A Supplementary data

Supplementary material 1 Image 1

Supplementary material 2 Image 2

Supplementary material 3 Image 3
