# Supplementary material for: Adaptation of utility functions to reward distribution in rhesus monkeys
Source: Cognition. 2021 Sep;214:104764. doi: 10.1016/j.cognition.2021.104764 (PMC8346953; doi:10.1016/j.cognition.2021.104764)
Supplement: Supplementary file 1 — Supplementary material 1 [file mmc1.docx]

**Raw, single-trial choice data for**

**Adaptation of utility functions to reward distribution in rhesus monkeys**

by

**Philipe M. Bujold, Simone Ferrari-Toniolo, Wolfram Schultz**

The dataset contains >125,000 single trials, capturing the choices of 3 monkeys spread over months. Not all trials present in the dataset were used in the final elicitation of utility (some blocks were removed as they did not follow the fractile step-by-step order). All trials are risky versus safe decisions, imperative and safe versus safe trials have been removed from the dataset. Variables that require definitions - *monkey*: T73 is monkey A, T68 is monkey B, and U74 is monkey C; *trialNo*: the identifier of an individual trial during a day of testing. *gambleA/B:* Magnitudes (in ml) and probabilities of a given reward presented to the monkeys. GambleA was on the left-side of the screen, gambleB on the right. Safe options are represented by [magnitude, probability, 0, 0]; two-options gambles are defined as [magnitude1, probability1, magnitude2, probability2]; *outcomesCount:* How many individual rewards possible for [gambleA, gambleB]. 1 is a safe option, 2+ is a gamble, 0 no option appears; *GA_ev/GB_ev:* The expected values of the options presented in the position of gambleA and gambleB; *trialSequenceMode:* Software code identifying the sequence type that was run. 9050 mean fractile sequences generated automatically. 9020 are sequences read a pre-defined order file (define as variable sequenceFilename), and 9001 are sequences that are manually defined per block; *J_firstStimulus*: Time from stimulus appearance to first movement of the joystick; *J_onStimulus:* How long the joystick was held on stimulus during choice.
