# Supplementary material for: Adaptation of utility functions to reward distribution in rhesus monkeys
Source: Cognition. 2021 Sep;214:104764. doi: 10.1016/j.cognition.2021.104764 (PMC8346953; doi:10.1016/j.cognition.2021.104764)
Supplement: Supplementary file 3 — Supplementary material 3 [file mmc3.docx]

**Supplementary material**

**Reliability of employed discrete choice model**


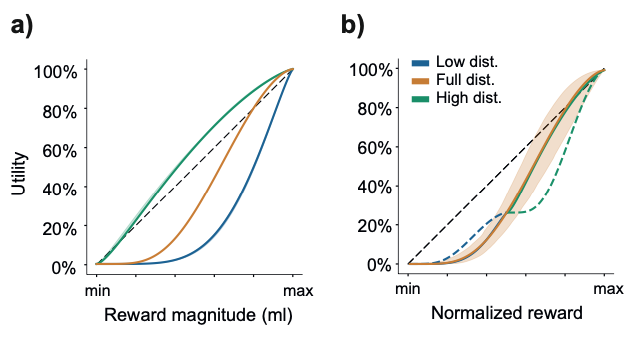


Figure S1. Validating the reliability of the U_2-Prelec_ discrete choice model (DCM) in capturing non-adaptating preferences. a) Scaled utilities estimated from simulated non-adapting preferences. Each curve represents the median of daily, distribution-specific parameters estimated from choices that were simulated using the median U_2-Prelec_ parameters of the monkey’s full-distribution utility (i.e., assuming monkey’s had non-adapting preferences). Choices were simulated using monkey B’s median full-range utility function for each daily elicitation sequence of the low- and high-reward distributions (Monte Carlo simulations, n=1000 for each daily session). Each simulated session had identical trials options and order to those used to elicit low- and high-distribution utilities. 95% Confidence intervals were estimated via boostrapping the median parameter estimate for each day (random sampling with replacement, n=10000). Dotted blue lines represent predictions full-distribution utilities predicted to fully-adapt to low-distributions. The dotted green lines represent similar full-adaptation predictions in the high distribution. The SAC score for the low-distribution was 1.44, that of the high-distribution was -0.33. b) Normalized utilities estimated from simulated non-adapting preferences. Again, curves represent the median of daily, distribution-specific parameters estimated from simulated choices, but normalized according to the minimum and maximum rewards in the tested distribution. Again, 95% confidence intervals were estimated via boostrapping (random sampling with replacement, n=10000). The GAC score for the low-distribution was 0.01, that of the high-distribution was 0.10.
